# Topographic Markers Drive Proteinopathies to Selection of Target Brain Areas at Onset in Neurodegenerative Dementias

**DOI:** 10.3389/fnagi.2018.00308

**Published:** 2018-10-05

**Authors:** Carlo Abbate

**Affiliations:** Geriatric Unit, Fondazione IRCCS Ca' Granda, Ospedale Maggiore Policlinico, Milan, Italy

**Keywords:** Alzheimer's disease, dementia, protheinopathy, neurodegeneration, adult neurogenesis, neural migration, cortical arealization, brain malformations

## Introduction

Neurodegeneration does not randomly hit the brain in degenerative dementias. Instead, it has relatively precise as well as consistent target regions over the brain at onset and an early stage of disease (accuracy and consistency of targeting). For example, pathology usually involves transentorhinal and entorhinal regions first in typical Alzheimer's disease (AD) (Braak and Braak, 1991). Moreover, data suggest that degenerative dementias also progress in a stereotypical topographic manner (Braak and Braak, 1991; Braak et al., 1993). However, what does it account for accuracy and consistency of targeting in degenerative dementias? More specifically: why does dementia start in that brain area where it effectively starts? Also, how can the diseases (i.e., proteinopathies) causing degeneration do to select their target brain area at dementia onset? In this article, I'll try to respond to these open questions about the onset of dementia, both considering the view by a current theoretical paradigm and presenting with a new hypothesis. The clinical progression of dementia and the underlying spread of degeneration over the brain are not topic of this paper. The discourse will be focused mainly on AD for clarity of exposition.

## The large-scale network paradigm and the problem opened by phenotypic diversity and clinico-anatomical convergence

Network theory assumes that neurodegeneration hits the brain and spreads along distinct large-scale neuronal networks, which are specific for each type of dementia (Seeley et al., 2009). Moreover, the concept of molecular nexopathy has been proposed to explain how each proteinopathy can select its definite large-scale network (Warren et al., 2013). In particular, molecular nexopathy refers to specific, coherent conjunctions of pathogenic proteins and intrinsic network characteristics, so that a large-scale neural network would manifest a selective vulnerability to a specific proteinopathy, by its cytoarchitecture, connectivity, or peculiar function. However, this exclusive relationship between a disease and a neural substrate seems to be contradicted from the evidence of phenotypic diversity and clinicoanatomical convergence in dementias (Seeley, 2017). In particular, phenotypic diversity refers to the fact that degenerative dementias appear quite heterogeneous in clinical manifestations. For example, it is well known that AD, beyond the classical and most frequent amnesic phenotype, may sometimes present with different focal syndromes (Lam et al., 2013). However, accuracy and consistency of targeting are valid also for the atypical syndromes of AD. Another type of phenotypic diversity is that some dementias may strike a brain area on the left hemisphere in a first patient and at the same time, the homolog brain area on the right hemisphere in a second patient (e.g., the left/right temporal variant FTD; Kumfor et al., 2016; Landin-Romero et al., 2016). Clinicoanatomical convergence refers to the fact that degeneration sometimes involves the same brain area in different dementias. For example, posterior cortical atrophy (PCA) syndrome can be associated indifferently with AD, corticobasal degeneration, Lewy bodies disease, as well as a not degenerative disease (e.g., Creutzfeldt-Jakob disease) (Crutch et al., 2017). A possible solution to the paradox of syndromic diversity (Warren et al., 2012a) has been offered by the concept of differential network disintegration (Warren et al., 2012b). However, as some authors underlined, some critical questions remain about which pathological proteins may account for connectivity disruption and which factors drive such differential network degeneration in AD syndromes (Bergeron et al., 2016).

## The hypothesis of a topographic markers system (TMS)

Bearing in mind the problem opened by phenotypic diversity and clinicoanatomical convergence, I believe that any hypothesis based on two actors only (i.e., proteinopathy and neural substrate) cannot quickly answer to the questions about the onset of degenerative dementias. Thus, I made a different hypothesis, which involves three actors. In particular, I hypothesized that selection of target brain areas in dementia would be regulated by a system which specifies topographical coordinates over the brain. This system would contain information about brain topography, probably at a quite macroscopic level of hemispheres, lobes, areas, gyres, which are activated when a proteinopathy interacts with the system in some way. Consequently, proteinopathies are driven by that information (i.e., topographic markers) to select their target brain areas. More plainly, it is the system that specifies where degeneration starts in the brain in this model. There would be not any particular relationship or affinity between proteinopathies and neural substrates (or networks), but the interaction between these two actors would be mediated by a third actor (i.e., a topographic markers system, TMS), that would be unique and independent from both proteinopathies and neural substrates.

## Neocortical arealization in development

**Figure 1:**
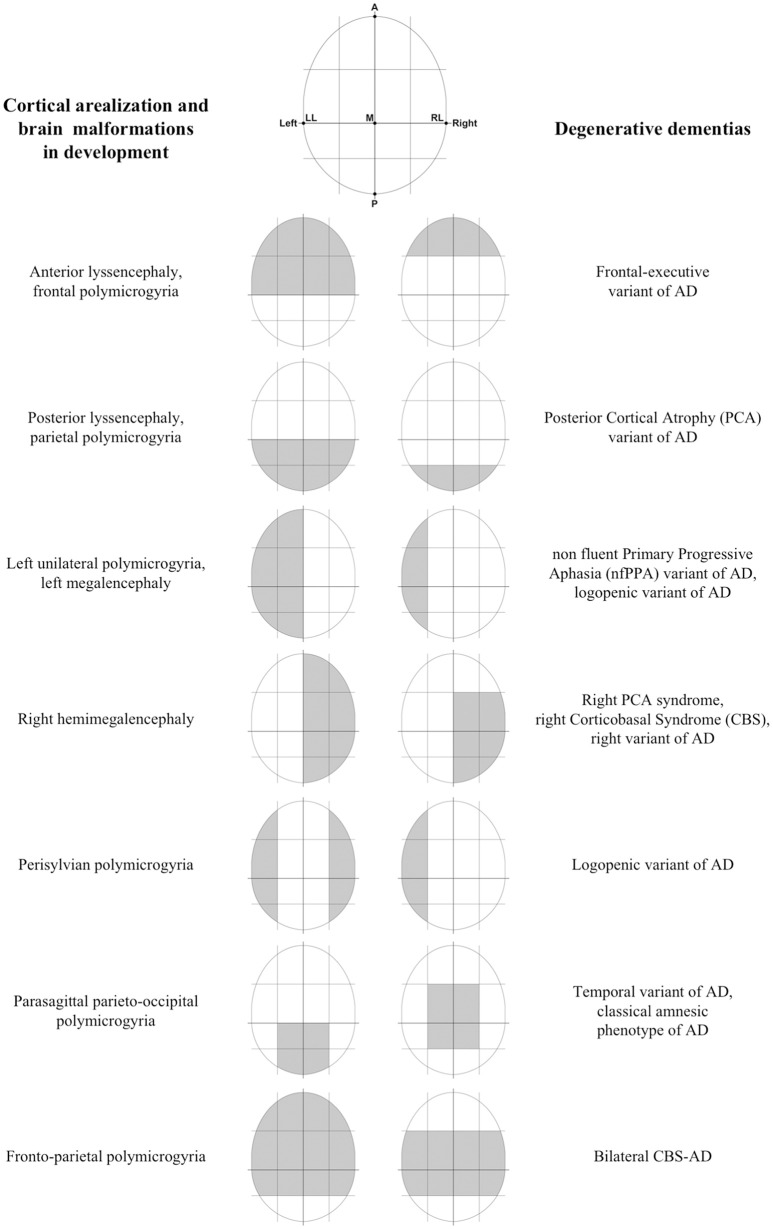
Some similarities between cortical arealization and brain malformations in development and degenerative dementias. Some preliminary findings of the mechanism controlling the progressive patterning of neocortical areas in development suggested that primary spatial information used are relating to simple brain axes. In particular, animal studies have demonstrated that there is an anterior-posterior (A-P) gradient of gene expression of morphogens or Transcription Factors (TFs), such that specific genetic factors enlarge rostral (motor) areas at the expense of caudal (sensory) areas, and vice versa (Chen et al., 2011). In addition to this A-P gradient, there is evidence for graded expression patterns along with other distributions, including the medial-lateral (M-L), and dorsal-ventral (D-V) axes. Interestingly, target brain areas at onset and early stages of degenerative dementias can be well and easily distinguished each other from the fact that involve different, and often opposite, locations along the same A-P, D-V, and M-L brain axes. In other words, there seem to be some similarities between the alphabet of spatial information about brain topography supposed involved in degenerative dementias and that used by the program of cortical arealization in development. Moreover, failures in some processes (e.g., abnormal cell proliferation, migration, and organization) during the development of the cortex have been associated with different developmental cortical malformations (Kanekar and Gent, 2011). The interesting feature here is that most malformations do not involve the entire cortex equally, but show regions of maximal severity. For example, some malformations (e.g., schizencephaly, megalencephaly, etc.) may involve alternatively one or both the hemispheres. A different type of malformation (i.e., lissencephaly) may present with two forms, one with maximal severity in the frontal lobes, and the other with maximal severity in the occipital lobes (Kanekar and Gent, 2011). Another more different malformation (i.e., polymicrogyria) shows a highly heterogeneous topographic distribution (e.g., frontal, frontoparietal, perisylvian, parasagittal parietooccipital, parietal, generalized), with a predilection for the perisylvian cortex (Leventer et al., 2010). At this regards, it is interesting to note that the distribution over the brain of some cortical malformations during development seems to be similar to the distribution of damaged brain areas in degenerative dementias at an early stage (LL, left lateral; RL, right lateral; A, anterior; P, posterior; M, medial).

The idea of a system which specifies information about macroscopic brain topography is not so surprising, considering that such system has been already found in a different research area, that is the morphogenesis of cortical areas in development. In particular, there is a complex mechanism which regulates the correct localization of the brain areas during development (i.e., cortical arealization; see Alfano and Studer, 2013), which necessarily uses some spatial information related to brain topography to work. This mechanism would be mostly under the genetic control of factors with discrete expression in the cortical field (protomap models). Interestingly, cortical arealization appeared to be highly conservative in its fundamental constituents among different clades of the mammalian class. For example, the reciprocal topology of primary areas along the anterior-posterior (A-P) and dorsal-ventral (D-V) axes of the neocortical surface remains fundamentally unaltered (see Alfano and Studer, 2013). This observation supports the role of a conserved genetic program orchestrating neocortical patterning which is inherited phylogenetically. Coming to degenerative dementias, I believe that the genetic program underlying cortical arealization in development would be a proper candidate to represent the TMS. Thus, I speculate that a re-activation of it would take place at a certain preclinical stage of degenerative dementias, and this leads to refreshing spatial information about macroscopic brain topography. Pathogenic proteins would interact with this information in some way and so would be driven to hit specific target brain areas. Interestingly, there seem to be some similarities between the alphabet of spatial information about brain topography used by the program of cortical arealization in development and that supposed involved in degenerative dementias (Figure 1). Moreover, considering that localization of brain areas follows a quite rigid scheme in mammalian, the accuracy, and consistency of targeting found in degenerative dementias are coherent with the view that proteinopathies would interact with the same scheme in some way.

## An unexpected corollary

How could the program of cortical arealization in development work in case of re-activation in a preclinical stage of degenerative dementias? A possible answer is based on the fact that two processes are surely implicated in cortical arealization, that are neurogenesis and neural migration. Accordingly, malfunction of these two processes is involved in many brain malformations in development (Kanekar and Gent, 2011). Not only, but the topographical function of the mechanism of arealization is also actualized overall through these two processes. Simplifying, in fact, brain areas are formed by newborn neurons which migrate from niches of neurogenesis toward exact locations over the brain, driven by complex signals (Alfano and Studer, 2013). This scenario leads to an unexpected corollary of the topographic markers hypothesis. It provides that neurogenesis and neural migration be probably implied in the onset of degenerative dementias. In this case, I speculate that diseases causing degeneration in dementias would begin in neural stem cells in the niches of adult neurogenesis. After that, the genetic program of arealization during development is re-activated, and the newborn neurons would receive some topographic instructions. Next, new “pathological” neurons would travel through the brain by neural migration and reach the target brain areas as specified by those instructions.

## Discussion

The idea that disease in adulthood would be based on reactivation of genetic programs active during development is not new, considering that has been already proposed for tumorigenesis (Reya et al., 2001; Naxerova et al., 2008) and heart failure (Taegtmeyer et al., 2010). Moreover, the influential role of adult neurogenesis in a brain disease has been already suggested for brain tumors (Vescovi et al., 2006; Sinnaeve et al., 2017). Also, a link between adult neurogenesis and AD has been already hypothesized. In fact, they share common sites where early pathology occurs, and newly-born neurons integrate into preexisting circuits (i.e., the hippocampal formation and olfactory bulb, OB) (De la Rosa-Prieto et al., 2016). Besides, some molecules are implicated in both the processes (e.g., APOE, PS1, APP, etc.; Lazarov and Marr, 2010; Mu and Gage, 2011; Hollands et al., 2016). Finally, animal studies suggest that APP can regulate neuronal migration in the developing cortex (Nicolas and Hassan, 2014). However, adult neurogenesis in humans is far to be a definitive finding (Sorrells et al., 2018). Moreover, some data suggest that neurogenesis and neural migration decline with aging (Spalding et al., 2013). On the contrary, the TMS hypothesis presupposes efficient neurogenesis and multiple migratory streams, especially to explain phenotypic heterogeneity in dementias. A possible reply is that the TMS may be reactivated many years before the clinical phase of dementia so that the selection of target areas in dementia would occur when neurogenesis and neural migration are still relatively efficient. The findings of the high prevalence of initial tau pathology in young people and its long pre-clinical presence seem coherent with this hypothesis (Braak and Del Tredici, 2011, 2015).

Another relevant aspect of the TMS hypothesis is that it is coherent with intracellular, more than extracellular pathology in dementia. So it agrees with data suggesting that TAU pathology is a primary event in the development of AD (Brier et al., 2016; Shi et al., 2017).

The accuracy of targeting in degenerative dementias is assured in TMS hypothesis by reactivation of a specific set of topographic information. Moreover, independence of TMS from both diseases and neural substrates opens to simple explanations for phenotypic diversity and clinicoanatomical convergence. In the first case, it is enough to hypothesize that different sets of topographic coordinates are reactivated in patients with same dementia. In the second, that the same set is reactivated in patients with different dementia, including those of not-degenerative etiology (e.g., prion disease). The consistency of targeting seems more challenging to explain. Why, in fact, a unique system as the TMS should consistently reactivate a different and specific set of topographic information for each dementia, so that distinct and typical brain areas are targeted in each one? A possible interpretation starts from the evidence that different dementias have different and typical ages of onset. At the same time, the functionality of the TMS probably modifies with aging. In particular, in young adults, some, or multiple, niches of neurogenesis may be still relatively active (e.g., the subventricular zone of the lateral ventricles) and long migrations, especially toward various regions on dorsal cortex, may be possible (see in adult macaques, Gould et al., 1999). In older adults, neurogenesis drops and migration may be limited to other few and different pathways (e.g., the rostral migratory stream toward the OB). Finally, in elderly, a unique niche of neurogenesis (e.g., the hippocampal subgranular zone) may remain active, and migration would be limited to short travels inside the hippocampus. Consequently, we wait for TMS selects different brain areas as the target, depending on the age of the patient. Accordingly, early onset AD frequently has regions on dorsal cortex as target areas, whereas late-onset AD is overall characterized by early medial temporal involvement (van der Flier et al., 2011). In sum, I speculate that dementias have different typical target areas at the onset because the functionality of a common mechanism of selection of target brain areas changes with aging, more than because the underlying diseases are different.

Considering that after brain injury in adults, neurogenesis increases (Yu et al., 2008) and neural migration redirects toward the site of lesion (Kaneko et al., 2017), TMS can also explain the peculiar localization of pathological tau found in traumatic degenerative dementia (Chronic Traumatic Encephalopathy). In fact, it is in perivascular regions and depths of sulci, that are the most stressed sites (McKee et al., 2016; Vile and Atkinson, 2017).

Finally, an appeal of the TMS hypothesis is that it proposes a unique mechanism of target areas selection for different neurodegenerative dementias. So, a full understanding of such mechanism might leave open the possibility to intervene on it, to block, deviate or suitably target neurodegeneration in different dementias.

## Author contributions

The author confirms being the sole contributor of this work and has approved it for publication.

### Conflict of interest statement

The author declares that the research was conducted in the absence of any commercial or financial relationships that could be construed as a potential conflict of interest.

## Abbreviations

TMS: topographic markers system.
